# P368: The economic assessment of an environmental intervention: discrete deployment of copper for infection control in ICUs

**DOI:** 10.1186/2047-2994-2-S1-P368

**Published:** 2013-06-20

**Authors:** M Taylor, S Chaplin

**Affiliations:** 1 York Health Economics Consortium, York, UK

## Introduction

Health Economics evaluations are typically applied to medications or surgery costs, but this unique study has investigated the economic benefits of discrete deployment of antimicrobial copper alloy touch surfaces in ICUs.

Copper/copper alloy surfaces have been shown to act as an adjunct to standard infection control practices in diverse clinical settings, continuously reducing contamination by over 90%. Moreover, work reported by Dr Michael Schmidt at the first ICPIC revealed the link between reduced bioburden and significant reductions in the risk of acquiring an HCAI.

This study investigates the cost-effectiveness of this intervention, comparing expenditure with the improvements in patient outcomes and other tangible benefits.

## Methods

Following an extensive literature review and use of expert opinion a number of factors have been considered in this evaluation, including component cost and longevity balanced with cost of care. Despite a lack of robust comparable data on the cost of HCAIs available in the public domain, good references were identified and used to calculate cost of care. Commercial data is available for the cost of the intervention and has been used to predict a return on investment (ROI) for installing a set of copper components as part of a new build or planned refurbishment. Consideration is also given to who in a hospital might specify such an intervention, where the budget resides and where cost savings could be realised. A model has been created to show the economic impact of an environmental intervention.

## Results

The model predicts the cost of replacing key, frequently-touched surfaces in a 20-bed UK ICU with copper equivalents will be recouped in less than two months. Thereafter, ongoing cost savings will accrue from the reduction in blocked beds and better-directed staff resources.

## Conclusion

The investigation allowed the derivation of a spreadsheet-based model that uses the best current published information and shows the rapid ROI of a copper intervention. It also calculates the impact on bed days and quality-adjusted life years (QALY). The model is simple, transparent to those with knowledge of spreadsheets, and allows adaptation to specific local settings.

## Disclosure of interest

None declared

